# Trends in Digital Twin Framework Architectures for Smart Cities: A Case Study in Smart Mobility

**DOI:** 10.3390/s24051665

**Published:** 2024-03-04

**Authors:** Evanthia Faliagka, Eleni Christopoulou, Dimitrios Ringas, Tanya Politi, Nikos Kostis, Dimitris Leonardos, Christos Tranoris, Christos P. Antonopoulos, Spyros Denazis, Nikolaos Voros

**Affiliations:** 1 ECE Department, University of Peloponnese, 26334 Patras, Greece; hristope@ionio.gr (E.C.); riggas@ionio.gr (D.R.); tpoliti@upatras.gr (T.P.); ch.antonop@uop.gr (C.P.A.); voros@uop.gr (N.V.); 2 Department of Informatics, Ionian University, 49132 Corfu, Greece; 3 ECE Department, University of Patras, 26504 Patras, Greece; sdena@upatras.gr; 4 Yodiwo, 26441 Patras, Greece; nk@yodiwo.com (N.K.); dl@yodiwo.com (D.L.); 5 P-NET New Generation Emerging Networks & Verticals, 26504 Patras, Greece; ctranoris@p-net.gr

**Keywords:** digital twin, smart mobility, smart cities, metacities, smart parking, urban mobility

## Abstract

The main aim of this paper is to present an innovative approach to addressing the challenges of smart mobility exploiting digital twins within the METACITIES initiative. We have worked on this issue due to the increasing complexity of urban transportation systems, coupled with the urgent need to improve efficiency, safety, and sustainability in cities. The work presented in this paper is part of the project METACITIES, an Excellence Hub that spans a large geographical area, that of Southeastern Europe. The approach of the Greek innovation ecosystem of METACITIES involves leveraging digital twin technology to create intelligent replicas of urban mobility environments, enabling real-time monitoring, analysis, and decision making. Through use cases such as “Smart Parking”, “Environmental Behavior Analysis on Traffic Incidents”, and “Emergency Management”, we demonstrate how digital twins can optimize traffic flow, mitigate environmental impact, and enhance emergency response; these use cases will be tested on a small scale, before deciding on implementation at a larger and more expensive scale. The final outcome is the METACITIES Architecture for smart mobility, which will be part of an Open Digital Twin Framework capable of evolving a smart city into a metacity.

## 1. Introduction

In the contemporary landscape, the confluence of digital technology, disruptive innovation, and urban/rural environments has given rise to the paradigm of smart cities and regions. Positioned at the forefront of this transformative wave, cities are evolving into ‘smarter’ entities by accelerating the adoption of digital technologies across diverse sectors. The integration of 5G, with the imminent arrival of 6G, alongside Big Data, sensors and IoT, AI, and Cloud Computing, has become intrinsic to a city’s digital infrastructure, intricately woven into the fabric of daily life and fundamentally altering societal dynamics in unprecedented ways. However, as the architectural and business models for building smart cities and regions continue to evolve, novel trends like digital twins and the metaverse are ushering in new dimensions to the discussion.

Yet, amid this technological leap, challenges emerge, marked by heightened fragmentation, complexity, and heterogeneity. The task at hand for cities and regions is to seamlessly adopt, deploy, and operate digital infrastructures and services amidst this intricate tapestry. Smart cities have metamorphosed into systems of systems, shaped by diverse drivers and evolving trends, including political, cultural, and economic factors. The technical challenge lies not only in integrating diverse technologies, platforms, and infrastructures, but also in presenting them in a user-friendly manner to cooperatively achieve common socio-techno-economic objectives.

Addressing these challenges necessitates a paradigm shift, deepening our understanding of urban dynamics and the impact of decisions made across cities. To this end, there is a growing imperative to create accurate and reliable reference representations of all entities—animate or inanimate, human or non-human—capturing their behavior, interactions, and information flows within the city and regional environment. A representation that constantly evolves through time and space, a living replica, is a digital twin.

Digital twins for smart cities empower stakeholders to assess the effects of changes before strategic plans are implemented [1,2]. Interconnected with the physical environment through digital infrastructures comprising sensors, wireless and optical networks, and cloud computing, digital twins facilitate the transfer, filtering, and processing of vast volumes of information. This information is utilized by sophisticated computer models to validate choices, simulate different scenarios, and identify strengths and weaknesses before deployment. Furthermore, Digital Twins enable real-time interactions with users, allowing direct control over city operations [3].

As a result, the forthcoming era of city and regional development, coupled with digital transformation, necessitates an approach that involves concurrent planning, construction, and operation of both the digital and physical facets of a city. METACITIES [4,5] envisions a strategic direction to establish an Excellence Hub, supported by a novel framework and a pioneering digital agenda. This approach harnesses the potential of emerging technologies, empowering interconnected entities, including companies, research institutions, governmental bodies, and societal actors, to mutually reinforce one another and collectively elevate the landscape of innovation in smart cities and regions.

METACITIES acknowledges the emergence of a nascent reality that has yet to achieve technical, scientific, organizational, and administrative maturity. Within this context, numerous opportunities unfold, providing the necessary impetus across various dimensions [6,7,8]. By investing in this emerging reality, particularly in digital twins, there is a substantial increase in the likelihood of establishing an effective innovative ecosystem that can leverage the region’s potential and spearhead advancements in this field. The resulting knowledge, innovative projects, and new relationships forged stand as pivotal assets, laying the groundwork for the sustained future of the region.

The main aim of this article is to present our innovative approach to addressing the challenges of smart mobility exploiting digital twins within the METACITIES initiative and especially the METACITIES Architecture for Smart Mobility. This architecture will be part of our proposal for an Open Digital Twin Framework (ODTF) that will facilitate the co-development of complementary future city domains with strong social footprint along with their corresponding DTs, like smart mobility. In the Background section, we present the significance of digital twins in smart cities and a number of case studies focusing on smart mobility. Following that, we describe the areas of smart mobility in which the Greek innovation ecosystem of METACITIES will leverage digital twin technology and specifically the use cases of “Smart Parking”, “Environmental behavior analysis on traffic incidents”, and “Emergency Management”. Then, the requirements and specifications that will guide us to the development and design of the architecture are presented. Finally, we propose the METACITIES Digital Twin Architecture, which will be able to support the evolution of a smart city to a metacity.

This article presents a new digital twin architecture that constitutes a common platform for collaboration and best practices, sharing across borders, sectors, and disciplines of knowledge production, circulation, and use. These multi-domain aspects and the focus on the application domain of smart cities is not addressed by the state of the art and the existing literature and standards (e.g., ITU T-REC-Y.3090 [9] and ISO 23247 [10]). On the other hand, our proposed digital twin platform is instantiated in three location-based innovation clusters in the southeastern European region (GR-CY-BG) and consists of a new framework that exploits the new enabling technologies in smart cities. The requirements of the platform presented in this article were derived from focus groups and show the Greek aspect of smart mobility. Additionally, our approach aims to enable new and improved user experiences with novel smart city products and services and understand how data services can be used to provide better service experiences with guaranteed quality of service.

This paper is organized as follows. Section 2 presents a literature review about the smart mobility state-of-the-art solutions and architectures. Section 3 describes the smart mobility use cases that the proposed architecture and the requirements are based on. Section 4 presents the proposed digital twin architecture and requirements, and Section 5 concludes the paper.

## 2. Background

Digital twins emerged as a prominent inclusion in Gartner’s top 12 strategic technology trends for 2022, as part of autonomic systems [11]; projections suggest that *“By 2027, over 40% of large organizations worldwide will be using a combination of Web3, spatial computing and digital twins in metaverse-based projects aimed at increasing revenue”* [12]. The World Economic Forum’s release of an insightful report on the framework and global practices for Digital Twin Cities [13] and the National League of Cities’ report on Cities and the Metaverse [14] underpin the significance of digital twins as a pivotal element in smart cities. These reports underscore how digital twins facilitate the development of virtual replicas of physical assets, sensors, systems, and processes. Moreover, they highlight the potential transformative impact of digital twin frameworks on smart cities, envisioning a seamless integration between the physical and digital realms.

### 2.1. Digital Twin Definition

The year 2002 saw the birth of the digital twin concept, within the Mirrored Spaces Model [15], envisioning digital models created in virtual space interacting with physical objects throughout their lifespan. This initial idea was solidified in 2012 with a formal definition; a digital twin [16] is a comprehensive virtual reflection of a physical entity, incorporating data like simulations, sensors, and historical information. This mirrored representation provided insights into the entire life cycle of the physical product.

Fast forward to 2018, and the concept took a giant leap; the “digital twin city” emerged [6]. Leveraging information technology, this approach builds a digital replica of a real city, complete with holographic simulations, dynamic monitoring, real-time diagnosis, and accurate predictions. By virtualizing all urban elements, it opens doors to intelligent and collaborative management of the entire metropolis. Physical and virtual cities can even interact and operate in parallel, further enhancing efficiency. To take it a step further, the “smart city digital twin” [7] envisions an intelligent, data-rich platform replicating and simulating real-city changes. This platform, powered by the Internet of Things, aims to optimize resilience, sustainability, and ultimately, the overall livability of our cities.

### 2.2. The Power of Digital Twins in Building Smarter Cities

Since 2019, digital twins have become a game-changer for smart cities. Imagine a virtual replica of a city that constantly learns and evolves, providing real-time insights and supporting smarter decision making in everything from urban planning to citizen engagement. That is the power of digital twins. Here, we elucidate how digital twins contribute to the progression of smart cities [17,18,19,20,21].

- Integration of IoT and Big Data:
  Embedded sensors in urban infrastructure feed data on energy, traffic, air quality, and more. Big data analysis uncovers patterns and trends, leading to improved sustainability, resource management, and service delivery [22,23].
- Real-Time Monitoring and Predictive Analysis:
  Digital twins monitor a city in real time, predicting future conditions like infrastructure issues or traffic jams. This proactive approach allows for preventative maintenance and resource allocation, ensuring smooth operation [24,25].
- Urban Planning and Design:
  Simulate the impact of new projects or policy changes before investing. Test-drive virtual designs to assess traffic flow, energy consumption, and environmental impact, optimizing design and minimizing costly mistakes [26,27].
- Improved Operational Efficiency:
  Predictive maintenance becomes possible through continuous monitoring and real-time data analysis, identifying potential equipment failures before they occur. This reduces downtime and saves costly repairs [28].
- Citizen Engagement and Participation:
  Interactive platforms powered by digital twins allow citizens to access real-time information, report issues, and provide feedback and leverage citizens’ engagement and participation. This fosters transparency and collaboration between citizens and city authorities [1,29].
- Resilience and Emergency Response:
  Simulate emergency scenarios like natural disasters or security threats and enhance cities’ resilience. Digital twins help cities understand potential impacts, optimize response plans, and allocate resources effectively.
- Data Security and Privacy:
  Trust and privacy are essential in smart cities, especially when it comes to data, so keeping data secure is paramount for digital twins. Advanced encryption, secure access controls, and anonymization techniques are all employed to protect citizen data and prevent unauthorized access. This way, residents can be confident their information is handled responsibly and used for the benefit of the city [18,30].
- Integration with Emerging Technologies:
  Digital twins are not just passive replicas; they are constantly evolving alongside cutting-edge tech. Artificial intelligence (AI) and machine learning (ML) supercharge data analysis, uncovering hidden patterns and trends for better decision making. Augmented reality (AR) adds an immersive layer, overlaying real-time data onto the physical city to visualize planning scenarios or guide maintenance crews.
- Interoperability and Standards:
  Open standards and frameworks are crucial for seamless integration between different digital twins and urban systems. Collaboration across organizations and industry consortia unlocks further innovation and progress, ensuring compatibility.

### 2.3. Digital Twin and Smart Mobility Paradigms

In the transportation and smart mobility domain, a number of digital twin studies have been undertaken very recently [31,32,33] focusing mainly on traffic management. In the following, we present some of the most characteristic digital twin paradigms for smart mobility, highlighting their benefits and positive outcomes.

#### 2.3.1. CityZenith DT

One notable use case that demonstrates the benefits of using digital twin technologies for smart mobility in the United States is the implementation of a smart transportation system in the city of Los Angeles, California [13,34]. Los Angeles has been actively deploying smart mobility solutions to address its traffic congestion challenges and enhance urban mobility. Using the digital twin, city officials can simulate and test different scenarios to identify potential bottlenecks, predict traffic congestion, and optimize traffic signal timings in real time. By continuing the CityZenith SmartWorld Pro platform, previously deployed in Chicago, the city has achieved notable benefits, including the following:

- Real-time Traffic Management:
  Los Angeles utilizes a network of sensors, cameras, and connected devices placed throughout the city’s transportation infrastructure to collect real-time data on traffic conditions. These data are processed using advanced analytics and AI algorithms to optimize traffic signal timings, manage lane directions, and dynamically adjust traffic flow. As a result, the city has experienced reduced congestion, improved traffic flow, and shorter travel times for commuters.
- Integrated Public Transportation:
  Los Angeles has integrated digital solutions into its public transportation system, including real-time transit tracking, electronic fare payment systems, and mobile applications for trip planning. These technologies allow commuters to access accurate information on bus and train schedules, track the arrival times of public transit vehicles, and plan their journeys efficiently. This integration has improved the overall reliability and convenience of public transportation, encouraging more people to use sustainable modes of travel.
- Parking Optimization:
  The city of Los Angeles has implemented smart parking systems that leverage sensors and mobile applications to provide real-time information on parking availability and guide drivers to vacant parking spaces. By reducing the time spent searching for parking, the city has reduced traffic congestion, enhanced urban accessibility, and improved the overall parking experience for residents and visitors.
- Data-driven Decision making:
  Los Angeles utilizes big data analytics and real-time data to make data-driven decisions regarding transportation planning and infrastructure investments. By analyzing traffic patterns, demand trends, and user preferences, city officials can identify areas of improvement, optimize transportation networks, and allocate resources effectively. These data are then fed into a digital twin, which is a virtual replica of the physical transportation system. The digital twin constantly updates itself based on the real-time data and provides an accurate representation of the current state of the transportation network. By combining this virtual model with advanced analytics and artificial intelligence algorithms, the city’s transportation authorities gain valuable insights into the system’s behavior and can make data-driven decisions to optimize traffic management.

The implementation of these smart mobility technologies in Los Angeles has resulted in several positive outcomes, including reduced traffic congestion, improved travel times, enhanced public transportation services, and optimized parking management. These advancements contribute to a more efficient and sustainable urban transportation system, making the city more livable and improving the overall quality of life for its residents.

#### 2.3.2. Barcelona 15-Minute City DT

In Europe, the city of Barcelona, Spain, has implemented as part of the 15-minute city initiative an innovative smart mobility system that integrates digital twins to optimize transportation networks and improve urban mobility [35]. By deploying a network of sensors, cameras, and IoT devices across the city’s transportation infrastructure, Barcelona collects real-time data on traffic conditions, parking availability, and public transportation usage. These data are then fed into a digital twin, a virtual replica of the city’s transportation system. The digital twin continuously updates itself based on the real-time data, allowing city officials to monitor the current state of the transportation network accurately. Advanced analytics and AI algorithms are applied to the digital twin to gain insights into traffic patterns, identify congestion hotspots, and predict future mobility challenges. Using the digital twin, Barcelona’s transportation authorities take proactive measures to optimize traffic flow and enhance mobility:

- Intelligent traffic management:
  The digital twin enables real-time monitoring of traffic conditions, allowing officials to identify bottlenecks and dynamically adjust traffic signal timings. By synchronizing traffic signals based on the current traffic flow, the city can reduce congestion and improve overall traffic efficiency.
- Parking optimization:
  The digital twin integrates data on parking availability and occupancy. This information helps city authorities direct drivers to available parking spaces and reduce the time spent searching for parking, resulting in reduced traffic congestion and improved urban accessibility.
- Public transportation enhancements:
  By analyzing real-time data on public transportation usage, the digital twin assists in optimizing bus and metro routes. The city can adjust schedules, allocate resources more efficiently, and provide accurate travel information to passengers in real time, improving the reliability and attractiveness of public transportation.
- Mobility planning and simulation:
  The digital twin serves as a valuable tool for city planners to simulate and evaluate different mobility scenarios. They can assess the impact of proposed infrastructure changes or policy interventions before implementation, ensuring more effective and informed decision making.

The successful implementation of this smart mobility system in Barcelona has resulted in several positive outcomes, like reduced congestion, improved traffic flow, increased utilization of public transportation, better parking management, etc.

#### 2.3.3. Singapore Vizzio DT

In Asia, the Singapore-based VIZZIO Technologies “cloned” all of Singapore to create the world’s biggest digital twin [28,36]; the 1:1 scale model of Singapore is divided into 1-square-meter titles, totaling 728,000,000. As part of this effort, Singapore has implemented a comprehensive smart mobility system that leverages digital twins to optimize transportation and improve urban mobility. With an extensive network of sensors, cameras, and IoT devices deployed across the city, Singapore collects real-time data on traffic conditions, public transportation usage, and various mobility aspects. These data are fed into a digital twin, a virtual replica of the city’s transportation system, to enable accurate monitoring and analysis. The digital twin integrates data from various sources and applies advanced analytics and AI algorithms to gain insights and enable smart decision making. Here is how this use case unfolds:

- Dynamic traffic management:
  The digital twin continuously monitors traffic conditions, including congestion levels, flow patterns, and incidents. By analyzing these data in real time, Singapore’s transportation authorities can dynamically adjust traffic signals, manage lane directions, and optimize road capacities to improve traffic flow and reduce congestion.
- Integrated public transportation:
  The digital twin integrates real-time data from buses, trains, and other modes of public transportation. This allows for seamless coordination of schedules, optimized route planning, and efficient resource allocation. Passengers can access real-time information, such as arrival times and service disruptions, through mobile apps, enabling a smoother and more reliable public transportation experience.
- Multi-modal trip planning:
  Leveraging the digital twin, Singapore provides a comprehensive multi-modal trip planning platform. Users can access mobile apps or websites that integrate real-time data from various transportation modes, including public transport, ride sharing, bike sharing, and walking. The digital twin analyzes these data to suggest the most efficient and sustainable routes based on individual preferences and real-time conditions.
- Predictive maintenance and safety:
  The digital twin enables predictive maintenance for transportation infrastructure, such as bridges and tunnels, by analyzing sensor data and identifying potential issues before they occur. This proactive approach helps prevent disruptions and ensures safe and reliable transportation for commuters.

The successful implementation of this smart mobility system in Singapore has led to several significant outcomes such as improved traffic flow, enhanced safety and security via incident detection, reduced environmental impact, etc.

In the next two tables, we present a comparison of these smart mobility paradigms, focusing on their features (Table 1) and their positive outcomes (Table 2).

Based on the provided comparisons, the desirable features of the smart mobility paradigms in the METACITIES initiative can be summarized as follows. Real-time Traffic Management involves the utilization of a network of sensors, cameras, and AI algorithms for continuous monitoring and optimization of traffic flow. Integrated Public Transportation ensures seamless coordination of schedules, optimized route planning, and real-time information for passengers to enhance the efficiency of public transportation systems. Parking Optimization integrates data to direct drivers to available parking spaces, reducing search times and congestion as well as enhancing environmental behavior analysis. Data-driven decision making is facilitated through the utilization of big data analytics for transportation planning and infrastructure development, enabling informed decision-making processes. Predictive Maintenance and Safety strategies are implemented to prevent disruptions in transportation infrastructure, ensure safety for commuters, and emergency management. Finally, Optimized Resource Allocation ensures efficient transportation management, leading to cost savings and improved resource efficiency through optimized allocation of resources.

In terms of outcomes, the METACITIES smart mobility paradigms aim to achieve a multitude of goals. These include Improved Traffic Flow, facilitated by real-time monitoring and data-driven decisions, contributing to smoother flow and reduced congestion. Enhanced Public Transportation is realized through the integration of digital twins, improving coordination and efficiency in public transportation systems. Moreover, Personalized Travel Recommendations are provided, offering tailored suggestions based on insights derived from data analytics. The initiative also prioritizes Increased Sustainability by reducing unnecessary vehicle kilometers traveled, lowering emissions, and improving air quality through sustainable transportation practices. Additionally, efforts are directed towards reducing congestion and shortening travel times, optimizing traffic flow, and encouraging modal shift to Sustainable Transportation options to mitigate environmental impact. Urban Accessibility is enhanced by improving accessibility within urban areas through efficient transportation management and infrastructure development. Furthermore, Predictive Maintenance and Safety strategies are implemented to ensure the safety and reliability of transportation infrastructure, alongside Optimized Resource Allocation, which results in cost savings and improved resource allocation. These features and outcomes underscore the comprehensive approach of the METACITIES initiative in addressing the challenges of smart mobility and fostering sustainable urban development.

## 3. METACITIES Use Cases for Digital Twin Smart Mobility

### 3.1. METACITIES Concept

METACITIES’ overall objective [4,5] is to establish an Excellence Hub for future cities and regions in Southeast Europe, built upon digital twins and metaverse technologies, through cross-border collaboration, focusing on innovation and engaging all different categories of actors of the quadruple helix. The three participating clusters, Cyprus, Greece, and Bulgaria, have selected domains with a strong social footprint as reference implementations in order to guide and validate the proposed framework.

The first set of challenges raised by METACITIES relates to the choice of the ICT infrastructures and platforms that should be deployed, interconnected, and operated across the city, ensuring that they have the capabilities and resources to realize our vision. The ICT infrastructures are not homogeneous since they encompass a diverse set of network, computation, and platform technologies, so they require careful planning and orchestration to meet future requirements. The second innovative technical dimension that facilitates the goal of creating, maintaining, and managing digital twins and metaverses for future smart cities is the role of the ODTF, Open Digital Twin Framework, a reference architecture that describes the digital model, function, and interactions of its physical counterparts. ODTF provides an integrated object framework for developers consisting of digital twin and metaverse building blocks that can be used to build domain-specific digital twins and metaverses enabled by the ICT infrastructures of the smart city. Also, well-known challenges from the literature review [1,2,17,18,19], include data privacy concerns, interoperability issues between different systems and platforms, the complexity of integrating diverse data sources, ensuring the accuracy and reliability of predictive models and the use of AI and big data, addressing the digital divide to ensure equitable access to digital twin technologies for all citizens, and addressing the high costs of implementation due to increased amount of sensors and computational resources as well as overcoming communication network-related obstacles.

Answering the aforementioned challenges, METACITIES will design pilots and demonstrators drawn from specific smart city sectors, namely, smart mobility, smart buildings and energy efficiency, and smart urban planning, jointly contributed by the three clusters (CY, BG, GR). These smart cities sectors have been selected as a result of the participating cities’ own interests and current investment plans that are in the pipeline. Specifically, the Greek innovation ecosystem of METACITIES involves leveraging digital twin technology for smart mobility; detailed smart mobility use cases are designed and presented in the following subsection. METACITIES will develop a number of prototypes by building instances of the proposed digital twins and metaverses according to the ODTF and using actual ICT infrastructures, which will act as proofs of concept, providing useful feedback for validating and improving the designs. In addition, a leading academic partner (Aarhus University) that is from a non-widening country (DK) is participating in the validation process, as its extensive experience on innovative business modelling will be capitalized upon to that effect. Aarhus University is also participating in a consulting role on technical (i.e., 5G/6G, smart cities, DTs) and innovative green business modelling and sustainability aspects.

**Figure 1:**
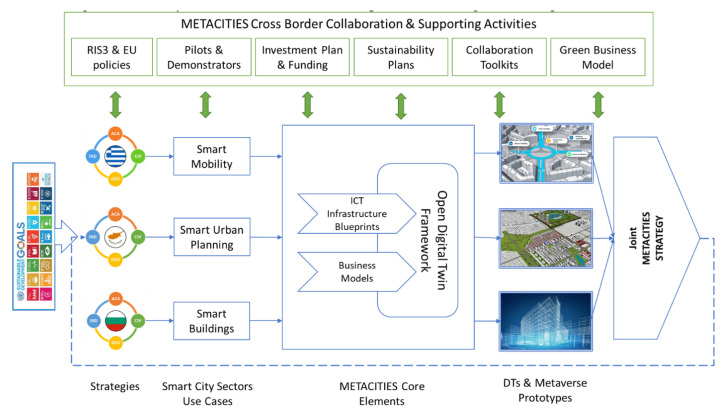
METACITIES—Cross-border collaboration and supporting activities.

The relevant methodological approach and the resulting action plan of the METACITIES project are depicted in Figure 1. They are oriented towards the way in which the proposed approach will be applied in order to achieve the objectives of the project. R&I strategies must be linked with goals that serve a greater purpose and bring prosperity and benefits to the society, e.g., the UN Sustainable Development Goals. It is therefore important that the proposed strategies and the accompanied measures have some kind of guarantees that will be successful before their actual implementation.

### 3.2. Use Cases for Smart Mobility

Traffic management challenges in urban areas are becoming increasingly critical, necessitating the modernization of outdated traffic control centers due to the escalating information load on operators. The complexity of these transport management systems intensifies with the growing number of people and modes of transport. Particularly, as the density of vehicles and pedestrians rises, the impact of minor incidents on traffic congestion becomes unpredictable, making swift and effective actions crucial for managing the substantial flow of vehicles and individuals.

The urgency of responding promptly to traffic incidents is paramount to minimize their impact on congestion. In economic terms, the annual cost of congestion in the EU has been estimated to be 1% of GDP [37]. Congestion also leads to negative externalities, including heightened levels of noise, pollution, increased potential for accidents, and a reduction in the useful life of vehicles and the network’s capacity to handle incidents and be resilient.

Moreover, according to the European Commission, the year 2020 saw 18,800 lives lost on European roads, translating to approximately 360 people tragically losing their lives every week [38]. Despite the extensive efforts of the United Nations and other road safety bodies over the past two decades, these alarming statistics have seen little improvement. Acknowledging the gravity of the issue and the imperative to address it, governments worldwide, through UN General Assembly Resolution 74/299, declared a Second Decade of Action for Road Safety 2021–2030, aiming to reduce road deaths and injuries by at least 50% during this period. Faster response times in the event of accidents can significantly reduce permanent disability and death rates [39].

These concerning statistics not only compromise safety and quality of life but also directly conflict with the environmental goals set by the METACITIES Smart City R&I strategy. Response time hinges not only on the timely identification and classification of incidents but also on the actions taken to effectively mitigate their influence. Hence, it is imperative for a modern Traffic Management system to be intelligent enough to predict the broader area’s incident impact and assist operators, law enforcement, and emergency services in executing an effective action plan.

**Figure 2:**
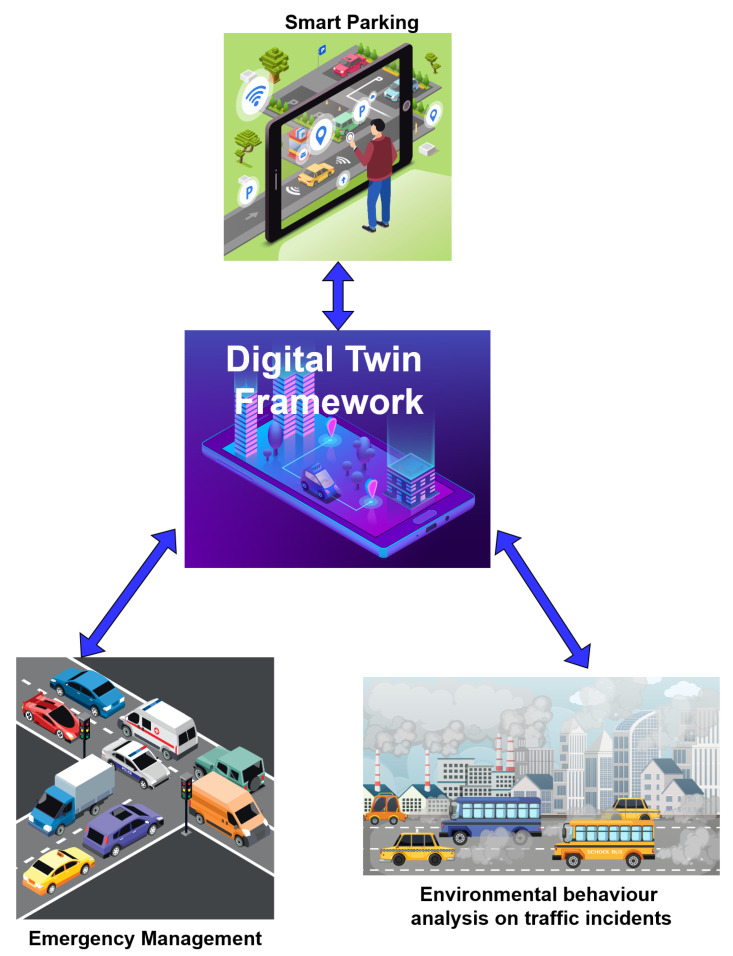
METACITIES—use cases for smart mobility.

The proof-of-concept experiments to be conducted on the digital infrastructure of the Municipality of Patras, Greece, will lay the foundation for the implementation of an Intelligent Transport and Incident Management System (ITIMS). According to METACITIES methodologies, policies designed to address transport issues in the City of Patras will rely on the creation of an efficient Smart Transport digital twin. This twin will monitor necessary data, providing a “testbed” for various policies and solutions before their actual implementation. The overarching goal is to facilitate the safe and smooth movement of vehicles, pedestrians, and bicycles through complex urban environments, aligning with the Smart Transport priorities for Safety, Quality of Life, Green initiatives, Accessibility, and Inclusivity goals set for the City of Patras. Specifically, METACITIES will leverage Digital Twin technology for the areas of “Smart Parking”, “Environmental behavior analysis on traffic incidents”, and “Emergency Management”, as shown in Figure 2.

- Smart Parking:
  By integrating data like parking availability, the system can provide real-time information on available parking spaces, evaluate Estimated Arrival, and suggest optimal routes to reach them. The digital twin will be able to visually exhibit the traffic situation and congestion in real time. Furthermore, the proposed digital twin should be able to run specific what-if scenarios in order to develop proposals for solving transport problems like optimization of traffic flow, pedestrian and cyclists’ flows, public transport alternatives, traffic management, parking management, etc. Also, it will suggest public transportation enhancements identifying areas with high demand.
- Environmental behavior analysis on traffic incidents:
  By integrating environmental data like air quality, noise, smoke, etc., and combining them with traffic data into the digital twin, the system can investigate the correlation that they have and the effect that traffic and/or incidents may have on the environment in a city of the city center. In METACITIES, the digital twin will be able to visually exhibit the environmental and noise situation and congestion in real time. Finally, an application will be designed that will interoperate with the digital twin and will be able to inform the stakeholders about possible eminent incidents that may compromise air quality.
- Emergency Management:
  Based on data and analysis collected and conducted in real time, the building blocks will be designed that will comprise an operating center for emergency services through which authorities can sync up their actions after a traffic incident. These actions include rerouting vehicles while facilitating emergency vehicles to access the incident when needed. At a later stage, this operating center will become an advanced notification system for drivers, cyclists, pedestrians, and public vehicles. It will also provide solutions to what-if scenario for optimization of traffic, optimization of traffic lights, and investment justification in the construction of transport infrastructure for emergency services (for example, extra lanes, etc.).

From the existing relative literature, it is clear that digital twins can drastically enhance the effectiveness of smart parking systems when correlated with monitoring traffic patterns, accidents, and driver/pedestrian behavior. Some key aspects the defined use cases will focus on are as follows. The first one is related to emergency situations, accidents, and how to prevent them or at least minimize them. Digital twins can analyze historical accident data within the parking area, identifying high-risk zones and accident patterns. This helps understand root causes and implement targeted safety measures like improved and multifaceted warning signaling, speed bump placement, or adjustments to parking layouts. Furthermore, real-time monitoring through the digital twin allows for faster detection and response to accidents. Data like vehicle behavior, movement pattern, speed, etc., or even pedestrian jaywalking leading up to the incident can be analyzed to identify possible causes and prevent similar occurrences in the future. The second use case highlighted in the paper is related to traffic management and congestion control. In that respect, visualization tools within the digital twin allow authorities to track individual vehicles, monitor traffic flow, and identify potential bottlenecks or congestion points in real time. This facilitates proactive measures like dynamic signaling or adjustments to traffic light timings based on specific conditions. By providing a holistic view of parking operations, digital twins enable companies but also public services to make data-driven decisions. Insights from historical data and real-time monitoring can inform future planning for parking infrastructure development, resource allocation, and policy implementation to better mitigate congestion and traffic city-wide. Finally, even the operation of the parking itself can be optimized by digital twin infrastructure continuously monitoring the health and performance of equipment within the parking area, such as parking sensors, lights, and gates. By analyzing sensor data and predicting potential failures, proactive maintenance can be conducted, minimizing downtime and disruptions. Additionally, from the commercialization point of view, the digital twin can also yield significant benefits through changes to the parking layout, pricing strategies, or traffic flow patterns. This allows for optimization of parking space utilization, revenue generation, and overall efficiency of the system.

### 3.3. Use Case 1—Smart Parking

Some of the main problems in the City of Patras that affects the quality of life, safety, and accessibility of various significant points of interest in the city center are the limited parking spaces and limited short-term parking solutions, large number of people living in the outskirts of the city, and bad parking behavior with respect to short-term parking. In order to propose solutions and policies that will overcome the problem of the illegal parking-induced congestion compromising the safety and the accessibility of cyclists, pedestrians, and drivers that visit or work at the city center, one needs to understand the problem.

In order to provide efficient and sufficient solutions, the first step is to monitor the traffic patterns and characteristics, various parking-related incidents, and driver/pedestrian behavior, in order to investigate and build an intelligent transport management system that may predict the effects of such illegal parking behaviors on the overall traffic and congestion. Hence, from the list of the existing sensors and data sources provided above, there are numerous combined and standalone data that can be used. The specific use case will deal with the Goal of Accessibility at the Smart City priority of Smart Transport.

*Input data sources and preconditions*

We will collect data from roadway sensors and cameras to study the impact of illegal parking behavior on traffic congestion. Such behavior includes, among other things, violations of restricted parking zones, double parking, and violation of maximum allowed parking time. Furthermore, crowd sourcing incident reporting may be sought to that effect. These conditions will be monitored and identified by advanced sensor networks with edge analytics.

At a second stage, we will investigate the effect of various policies on the traffic patterns and congestion, for example, we will seek the relation of the response time of traffic operators and municipal police and we will evaluate the impact of this time on traffic congestion.

At the final stage, various applications will be explored that will be able to interface with the digital infrastructure to provide various solutions to the smart mobility problems.

*High-Level Technical Approach*

In METACITIES, we will provide the design for the functional blocks and respective interfaces for a digital twin that will evaluate all the data mentioned in the previous section, combining traffic data, parking solutions and data, illegal parking behavior, and the effect that these will have on the traffic in the city center (e.g., a specific street). All the functional requirements will be defined and described.

The digital twin will be at the core of the Intelligent Transport and Incident Management System, which will in turn show in a visual way the traffic situation of specific roads.

In METACITIES, the digital twin will be able to visually exhibit the traffic situation and congestion in real time. Furthermore, the proposed digital twin should be able in the future to interface with functional models and algorithms that may be able to train the behavioral models so that it will also be able to run specific what-if scenarios in order to develop proposals for solving transport problems like optimization of traffic flow, pedestrian and cyclists flows, public transport alternatives, traffic management, parking management, etc. In METACITIES, the exact data flow, standards, and interfaces will be sought and defined.

Finally, the ultimate goal is to show how innovative applications will be interfacing with the DTs, for example, we will design an application that will interoperate with the digital twin and will ultimately be able to give stakeholders valuable real-time info with respect to the following:

- evaluate estimated arrival time combined with free parking slots in the area of interest for drivers;
- suggest alternative transportation/intermodal transport for the places of interest;
- provide alerts and perform incident reporting to first responders/police for incidents that may potentially turn into traffic blockage incidents.

### 3.4. Use Case 2—Environmental Behavior Analysis on Traffic Incidents

One of the main problems faced by big cities that compromises the quality of life of its citizens relates to the green dimension of city life. Especially for smart mobility, it has been identified as a compromising factor that needs to be dealt with for specific stakeholders, like cyclists and pedestrians, but also for all the citizens at large.

Monitoring environmental indexes is a very important factor when trying to apply policies and actions that will make a city greener, healthier, more sustainable, and more attractive for its citizens and visitors. The impact of traffic incidents on environmental indexes needs to be thoroughly analyzed by correlating traffic conditions with environmental (air quality, noise, smoke, etc.) measurements. The objective is for the primary focus of Smart Transport to transition toward being environmentally conscious and eco-friendly.

*Input data sources and preconditions*

By monitoring specific indexes through sensors and crowd source means, and making use of the air quality forecasting models already applied to the city of Patras, one can correlate and understand the expected environmental conditions after an incident at a certain city location. This may also involve footage from specific cameras and other data sources from the list in previous sections.

*High-Level Technical Approach*

In METACITIES, we will design the main functional blocks that will comprise a digital twin that will evaluate all the environmental (air quality, noise, smoke, etc.) data, and traffic data in order to investigate the correlation that they have and the effect that traffic and/or incidents may have on the environment in the city center, e.g., a specific street.

In METACITIES, the digital twin will be able to visually exhibit the environmental and noise situation and congestion in real time. However, the digital twin will be able to train air quality forecasting models and will be able to run specific what-if scenarios in order to develop proposals for improving the effect on the environment. The second use case will rely on the functional blocks that will assume a specific data flow from the sensors and data sources to the visualizations of the environmental issues in real time, while interfacing with environmental models. All elements will be described and designed.

Finally, we will design an application that will interoperate with the digital twin and will be able to inform the stakeholders about possible eminent incidents that may compromise air quality.

### 3.5. Use Case 3—Emergency Management

One main problem in large cities that affects the quality of life and the safety of the citizents relates to the road deaths and injuries that, through UN General Assembly Resolution 74/299—a Second Decade of Action for Road Safety 2021–2030, are set to be reduced by at least 50% [39]. It has become evident that for injuries, in the case of road accidents, shorter response times can significantly reduce people’s permanent disability and death rates. To that effect, an Intelligent Transport and Incident Management System (ITIMS) of a smart city should alleviate the issues of traffic congestion for emergency services.

*Input data sources and preconditions*

The data that are required will be similar to those mentioned in use cases 1 and 3. Data from from roadway sensors and cameras may be used for traffic prediction and blockage.

*High-Level Technical Approach*

Based on real-time collected data and analysis, we will design the building blocks, interoperability center, and interfaces that are required to be built for the digital twin that will comprise an operating center for emergency services through which authorities can sync up their actions after a traffic incident. These actions include vehicle’s rerouting while facilitating emergency vehicles to access the incident when needed. At a later stage, this operating center will become an advanced notification system for drivers, cyclists, pedestrians, and public vehicles.

Similarly to use case 1, data will be used to train traffic models that will enable quick decision making in the case of incidents that affect the traffic. The digital twin will be at the core of the Intelligent Transport and Incident Management System, which will in turn show in a visual way the traffic situation of specific roads as well as the transport network, and will develop proposals for solving transport problems. Specific solutions will be run as what-if scenarios when specific infrastructure changes are to be made.

It will also provide solutions to what-if scenarios for the optimization of traffic, optimization of traffic lights, and justification of investment in the construction of transport infrastructure for emergency services.

In Table 3 is depicted an overview of the key aspects of each use case, highlighting the addressed problems, data sources, technical approaches, and applications.

## 4. METACITIES Digital Twin Architecture

According to [13], the development of a digital twin city necessitates nine elements, encapsulating a “4 + 5” framework. The “4” denotes the four core elements, namely (1) infrastructure; (2) data resources; (3) platform capacity; and (4) application scenarios. These elements serve as the internal pillars, providing the internal power and digital infrastructure for the digital twin city. In contrast, the “5” in the framework encompasses the five key external supporting elements crucial for digital twin cities. These include (1) strategy and mechanisms; (2) stakeholders; (3) funding and business models; (4) standards and evaluation; and (5) cybersecurity.

Taking an abstract perspective, a digital twin for a smart city relies on various layers of data; these layers progressively incorporate information about the terrain, buildings, infrastructure, mobility, and IoT devices [8]. The digital twin utilizes data generated within the virtual smart city layer to conduct additional simulations. Subsequently, this information circulates through the layers of the model, ultimately influencing and implementing changes in the physical world.

### 4.1. Smart Mobility Requirements

Carrying out a comprehensive requirements’ analysis for the presented use cases on smart mobility, we conclude with a set of functional (Table 4) and non-functional requirements (Table 5).

Functional and non-functional requirements are two types of specifications that define what an application should do and how well it should perform. Functional requirements describe the actions and interactions that users can perform with the app, while non-functional requirements describe the quality attributes and features of the app. Smart mobility digital twins are designed to help people move around the city with ease and convenience, using innovative technologies and solutions. They provide real-time information on the best routes, schedules, and prices for different mobility options, such as buses, trains, cars, bikes, etc. But smart mobility applications are not only about convenience. They are also about quality and sustainability. They ensure that the mobility options they offer are user-friendly, reliable, secure, and scalable. They also encourage people to adopt more efficient and eco-friendly mobility behaviors, by providing feedback and offering incentives and rewards for choosing greener and smarter modes of transportation.

### 4.2. METACITIES Architecture for Smart Mobility

**Figure 3:**
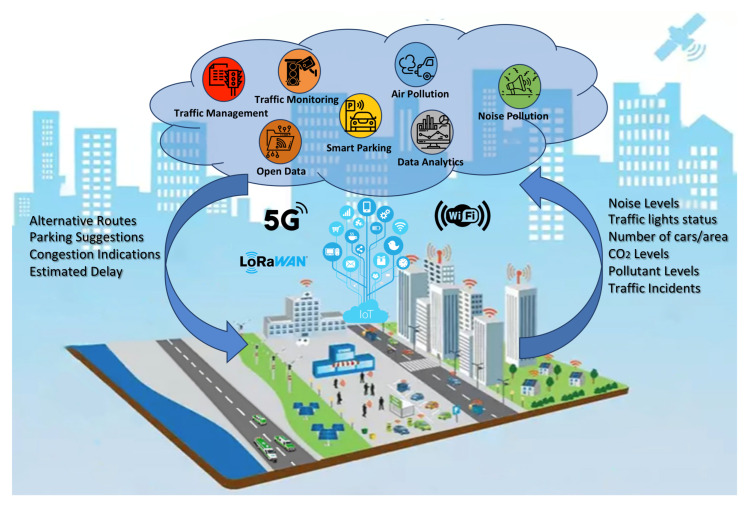
METACITIES—smart mobility.

**Figure 4:**
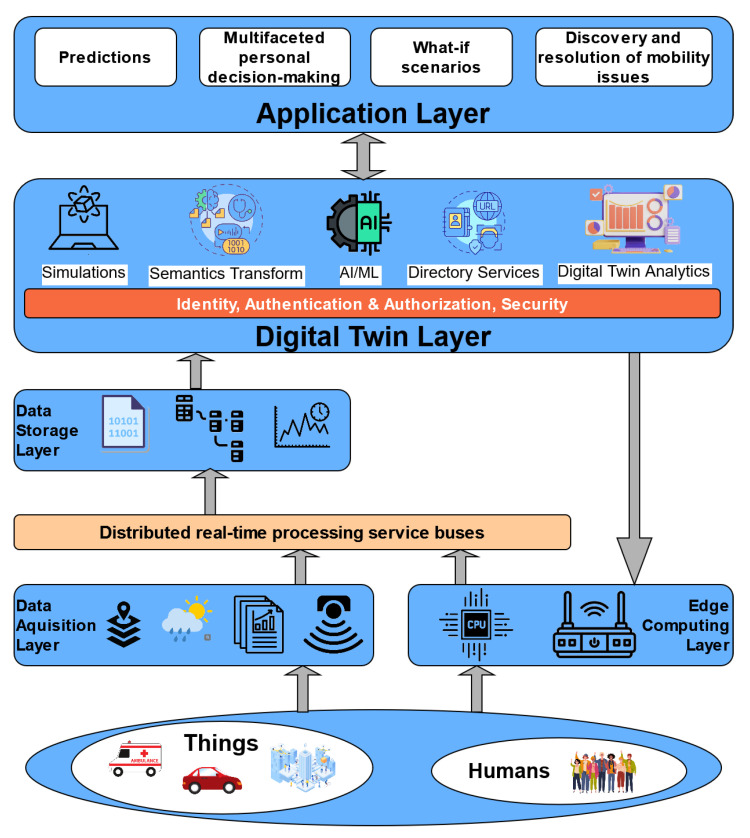
METACITIES—architecture for smart mobility.

Aiming to support all the use cases for Smart Mobility, as shown in Figure 3, we propose an overall architecture, as shown in Figure 4. The architectural diagram is high-level and depicts the initial blocks and requirements for the METACITIES architecture that can support the specific DT use cases. Layers are cleanly separated, with each serving clear purposes.

Data are collected from a number of things, like devices, IoT sensors, vehicles. as well as humans, e.g., via mobile apps for problem reporting and crowd sourcing and sensing. Additionally, data can be retrieved from simulators and field-use applications. The Edge Computing layer effectively handles actuation towards devices, allowing decisions made at the Digital Twin layer to reliably reach the devices. The Data Acquisition layer also handles reception of data from third-party services, using cloud-to-cloud service data such as weather, raw text/CSV, GIS and BIM, etc. Data from any sources are then fed into distributed real-time processing service buses that can perform filtering, routing, shaping, and processing of data before writing to the Storage layer.

The Storage layer handles all non-volatile storage needs and usually consists of specialized database management systems that cover different needs:

- Relational storage—SQL;
- Raw time-series storage for sensor data;
- Document storage;
- BLOB (Binary Large Objects) storage.

The Digital Twin layer is a series of microservices, closely (via IPC) or loosely (via Cloud APIs) interconnected between them. Those services carry the overall system intelligence, digital twin infrastructure, and AI/ML, and they rule execution subsystems and implement the decision-making outcome by issuing API requests towards the Edge Computing layers’ advertised API endpoints. This layer allows the system to use data processing and analytics services in order to derive insights and patterns from the data ingested previously via the acquisition layers. These services can include various algorithms, machine learning models, statistical analysis, and other data processing techniques to extract valuable information. Additionally, the digital twin architecture incorporates models and simulations that replicate the behavior of the physical and logical objects of the “real world”. These models allow the digital twin to predict future states, analyze what-if scenarios, provide recommendations, make decisions, actuate directly towards devices and third-party services, and create alerts.

Finally, the Application layer includes the user-facing applications (Web, Mobile, etc.) that enable consumption of analytics, guided what-if scenario execution, and all user interaction in general.

We believe that the approach of the METACITIES initiative and the proposed architecture could be very useful to the open audience, first of all, for taking into account factors such as regional dynamics, socio-economic considerations, and infrastructure challenges; this contextualization approach adds value by aiming to offer practical guidance and considerations tailored to a specific region. Additionally, we present real-world case studies of implementing digital twin technologies in the urban development context, which may provide valuable empirical evidence and practical examples that can support decision making and implementation strategies for similar projects. The adopted interdisciplinary, quadruple helix approach, engaging stakeholders from scientific, industrial, social, and public sectors, fosters a holistic understanding of the challenges and opportunities associated with digital twin implementation in urban environments and enhances the relevance and applicability of the findings to a diverse audience. While existing standards and frameworks may primarily focus on specific sectors such as manufacturing or networking, our approach offers insights into how digital twin concepts can be adapted and applied across different domains, including smart mobility, urban planning, transportation, energy management, etc. For example, the ITU T-REC-Y.3090 [9], also known as “Digital twin network – Requirements and architecture” focuses on Network-Specific DTs (i.e., DTNs) and their corresponding enablers (i.e., physical network components and architectures) and provides a generic framework. Similarly, the ISO 23247 [10] “Digital twin framework for manufacturing” addresses the requirements and challenges of digital twin deployment in manufacturing and outlines guidelines for creating digital twins of manufacturing processes, equipment, and systems and existing products. On the other hand, the METACITIES approach involves a variety of stakeholders, emphasizes interdisciplinary collaboration to address not only technical challenges but also social, economic, and policy aspects of digital twin implementation in urban environments and aims to tailor digital twin solutions to regions’ specific needs.

## 5. Conclusions

In conclusion, the transformative potential of digital twins in the context of smart mobility is a cornerstone within the METACITIES initiative. Digital twins emerge as a keystone technology, offering unparalleled capabilities to revolutionize the landscape of urban mobility. Smart mobility is envisioned as a dynamic and interconnected system, where digital twins play a central role in creating accurate and reliable replicas of various mobility aspects. The framework leverages digital twins to provide real-time information on parking availability, conduct environmental behavior analysis of traffic incidents, and facilitate efficient emergency management.

For Smart Parking, digital twins become instrumental in monitoring traffic patterns, incidents, and driver/pedestrian behavior. By creating a digital replica of the urban mobility environment, we may enable the prediction of the impact of parking behaviors on traffic congestion, contributing to the development of an intelligent transport management system.

In the case of Environmental Behavior Analysis on Traffic Incidents, digital twins facilitate the correlation of environmental indexes with traffic data. This capability empowers decision-makers to understand the effects of traffic incidents on the environment and transition towards environmentally conscious smart mobility solutions.

Within Emergency Management, digital twins serve as the core of an Intelligent Transport and Incident Management System. Digital twins can support visual exhibition of real-time traffic situations, enabling quick decision making for emergency services and proposing solutions through simulated scenarios.

The proposed architecture emphasizes that digital twins are not merely technological replicas but dynamic entities that constantly evolve through time and space. By seamlessly integrating with physical environments through advanced infrastructures, Digital Twins within the METACITIES initiative contribute to creating a smarter, more efficient, and resilient urban mobility ecosystem. This innovative approach fosters collaboration, innovation, and sustainability, paving the way for the next era of smart mobility in cities and regions.

The architecture presented is high-level and will be further enhanced, incorporating the findings from the pilot testing. The technical work carried out and technical choices made will be greatly fuelled by the non-technical aspects of the city. The architecture proposed will target a sustainable network which will have to reinforce the cross-border networking of the ecosystems’ actors while aligning regional smart specialization priorities and other strategic considerations. The system that will be based on the proposed architecture will serve as a common platform for collaboration and best practice sharing across borders, sectors, and disciplines on knowledge production and implement strategies and relevant investment plans.

Our future plans include designing the Open Digital Twin Framework, ODTF, which provides an integrated object framework for developers consisting of digital twin and metaverse building blocks, that can be used to build domain-specific digital twins and metaverses enabled by the ICT infrastructures of the smart city. We will also develop a number of prototypes by building instances of the proposed use cases according to the ODTF and using actual ICT infrastructures, which will act as proofs of concept, providing useful feedback for validating and improving the designs. Finally, we aim to exploit the proposed architecture and the designed ODTF in other sectors and disciplines, like green transport corridors and waterways for cargo, as both land-based and maritime transportation need intelligent infrastructure for the purpose of enhancing traffic safety and efficiency [40].

## Figures and Tables

**Table 1:** Smart mobility paradigms comparison—features.

Features	CityZenith DT (Los Angeles, CA, USA)	Barcelona 15-min City DT (Barcelona, Spain)	Singapore Vizzio DT (Singapore)
Real-time Traffic Management *	Network of sensors, cameras, and AI algorithms for traffic optimization.	Real-time monitoring and dynamic signal adjustments.	Continuous monitoring, real-time adjustments, and optimized road capacities.
**Integrated Public Transportation**	Real-time transit tracking, electronic fare payment, and mobile apps.	Real-time data optimization for bus and metro routes.	Seamless coordination of schedules and optimized route planning.
**Parking Optimization**	Smart parking systems using sensors and mobile apps.	Integration of parking availability data for reduced search times.	Integration of data for directing drivers to available parking spaces.
**Data-driven Decision making**	Big data analytics for transportation planning and infrastructure.	Real-time data used for optimizing bus and metro routes.	Real-time data on traffic conditions, public transportation usage, and various mobility aspects.
**Predictive Maintenance and Safety**	-	-	Predictive maintenance for infrastructure to prevent disruptions.
**Optimized Resource Allocation**	-	-	Efficient transportation management leading to cost savings and improved resource efficiency.

* First column highlights smart mobility features that are detailed per city.

**Table 2:** Smart mobility paradigms comparison—outcomes.

Outcomes	CityZenith DT (Los Angeles, CA, USA)	Barcelona 15-min City DT (Barcelona, Spain)	Singapore Vizzio DT (Singapore)
Improved Traffic Flow *	Real-time monitoring and data-driven decisions for smoother flow.	Real-time adjustments for improved traffic flow and reduced congestion.	Continuous monitoring for real-time adjustments and improved traffic flow.
**Enhanced Public Transportation**	Integration of digital twins improves coordination and efficiency.	Real-time data used for optimizing bus and metro routes.	Seamless coordination, optimized route planning, and real-time information for passengers.
**Personalized Travel Recommendations**	Potential for personalized travel suggestions based on insights.	-	Comprehensive multi-modal trip planning platform based on individual preferences.
**Increased Sustainability**	Optimized traffic flow and reduced congestion contribute to sustainability.	Reduction in unnecessary vehicle kilometers traveled, lower emissions, and improved air quality.	Efficient transportation management results in lower fuel consumption, reduced emissions, and improved air quality.
**Reduced Congestion**	Yes	Yes	Yes
**Shorter Travel Times**	Yes	Yes	Yes
**Modal Shift to Sustainable Transportation**	-	Yes	Yes
**Enhanced Urban Accessibility**	Yes	Yes	Yes
**Predictive Maintenance and Safety**	-	-	Predictive maintenance for infrastructure to prevent disruptions.
**Optimized Resource Allocation**	-	-	Efficient transportation management leading to cost savings and improved resource efficiency.

* First column highlights smart mobility outcomes that are detailed per city.

**Table 3:** Use Cases Overview.

Criteria	Smart Parking	Environmental Behavior Analysis on Traffic Incidents	Emergency Management
Problem Addressed *	Limited parking, illegal parking, congestion	Environmental impact of traffic incidents	Road deaths, injuries, and emergency response times
**Data Collection Sources**	Roadway sensors, cameras, crowd-sourced incident reports	Sensors, cameras, air quality forecasting models	Roadway sensors, cameras
**Data Analyzed**	Traffic patterns, parking incidents, driver/pedestrian behavior	Environmental (air quality, noise, smoke, etc.) and traffic data	Traffic conditions, emergency incidents
** Technical Approach**	Digital Twin for traffic management and incident prediction	Digital Twin for environmental and traffic data analysis	Digital Twin for emergency response and traffic management
**Real-time Visualization**	Yes	Yes	Yes
**Future Scenario Simulation**	What-if scenarios for traffic flow optimization	What-if scenarios for environmental impact mitigation	What-if scenarios for emergency response and traffic solutions
**Applications**	- Estimated arrival time and parking availability for drivers—Alternative transportation suggestions—Incident reporting	- Real-time environmental and noise situation visualization—Air quality forecasting models—Incident reporting	- Operating center for emergency services—Advanced notification system for road users—Traffic solutions and investment justifications
**Interfaces with Digital Twin**	Applications providing real-time information to users	Application informing stakeholders about environmental incidents	Operating center for emergency services

* First column highlights criteria that are detailed per use case.

**Table 4:** Functional requirements for smart mobility.

Use Case	Functional Requirement
Smart Parking	Real-time information on parking availability
	Integration with other transportation and urban planning systems
	Ability to work across different parking systems and providers
	Ability to work with multiple parking sensor vendors
	Ability to pay using different payment methods
	Ability to receive notifications about parking spot availability
	Ability to receive navigation instructions to available parking spots
	User-friendly interface
	Reminders about parking time expiration
	Automatic creation of parking tickets (fines) in cases of violating parking time expiration
	Ability to extend parking time remotely
	Ability to view parking history and receipts
	Showing location of handicap-accessible parking spots
	Automatically identify valid PwD (People with Disabilities) badge holders via use of Tags
	Issue parking ticket when vehicle without appropriate Tag parks in PwD spot
	Integration of mobile payment system
	Capability to manage parking availability and spaces through a digital platform
Emergency Management	Notifications to emergency services
	Identify type of emergency and number of injured persons
	Keep track of the available emergency vehicles and personel
	Real time dispatch of emergency services based on the severity of the incident
	Calculate shortest paths to the incident
	Assist traffic controllers to optimally reroute traffic to minimize disruption
	Automatically monitor traffic volume via appropriate sensors
Environmental Behavior Analysis on Traffic Incidents	Monitor air quality (pm rate)
	Measure noise
	Keep a record of environmental and traffic volume of time series data
	Correlate environmetal indices with traffic volume measurements

**Table 5:** Non-functional requirements for smart mobility.

Non-Functional Requirement	Smart Parking	Environmental Behavior Analysis on Traffic Incidents	Emergency Management
Privacy-preserving mechanism to safeguard information	✓	-	-
Protection of personal and financial information	✓	-	-
Accessibility and usability for people with disabilities drivers’ information	✓	-	-
Real-time parking availability monitoring using IoT sensors	✓	-	-
AI algorithms for predicting parking demand and optimizing space usage	✓	-	-
Data encryption and protection for security and privacy	✓	✓	✓
Data must be encrypted in-transit and at-rest	✓	✓	✓
All API endpoints must employ authentication and must mandate encryption	✓	✓	✓
Mobile application interface for accessing the smart parking digital twin	✓	-	-
Preference for a 2D interface for academia	-	✓	✓
Users must uniquely authenticate with the Digital Platform	-	✓	✓
Digital Platform must employ Role-Based Access Controls (RBAC) for logged in users	-	✓	✓

## Data Availability

Data are contained within the article.
